# Salmonella pyomyositis complicating sickle cell anemia: a case report

**DOI:** 10.1186/1752-1947-4-198

**Published:** 2010-06-30

**Authors:** Vanessa K Wong, Maxine E Lissack, Tom D Turmezei, Jenny A Maitland

**Affiliations:** 1 Department of Microbiology, Queen's Medical Centre, Derby Road, Nottingham, NG7 2UH, UK; 2 Department of Haematology, Mayday University Hospital, 530 London Road, Surrey, CR7 7YE, UK; 3 Department of Radiology, Queen's Medical Centre, Derby Road, Nottingham, NG7 2UH, UK

## Abstract

**Introduction:**

Pyomyositis is a bacterial infection of skeletal muscle and a rare complication of sickle cell anemia. It may present a difficult problem in diagnosis, leading to delay in appropriate treatment and development of complications including abscess formation and osteomyelitis.

**Case presentation:**

We report the case of a 44-year-old Afro-Caribbean woman with homozygous sickle cell disease who presented with chest crisis and later developed pyomyositis of her hip and pelvic muscles. *Salmonella agbeni *was isolated from blood cultures and magnetic resonance imaging confirmed the diagnosis in this case. It is noteworthy of this case that there were no antecedent signs of gastroenteritis. Drainage was not appropriate and she was treated with intravenous antibiotics for six weeks.

**Conclusions:**

Focal Salmonella infections are uncommon in soft tissue. Pyomyositis should be considered in patients with sickle cell anemia that continue to have muscle pain and high fevers, despite initial management of their sickle cell crisis. Radiological imaging, particularly magnetic resonance imaging, is a crucial tool in establishing the diagnosis.

## Introduction

Pyomyositis is a purulent infection of skeletal muscle that can rapidly lead to abscess formation [1]. It usually occurs in association with damaged muscle and impaired host defenses. Pyomyositis usually presents with fever and muscle pain, often in the lower extremity [2]. The most common causative organism is *Staphylococcus aureus*, which gives 'classic' pyomyositis [3]. The disease is a rare complication of sickle cell anemia with the diagnosis made even more difficult by the similarity of symptoms to a sickle cell crisis. We describe the case of a 44-year-old Afro-Caribbean woman admitted with sickle cell crisis who subsequently was diagnosed with Salmonella pyomyositis of her hip and pelvic muscles.

## Case presentation

A 44-year-old Afro-Caribbean woman missionary worker with homozygous sickle cell disease (HbSS), originally from Grenada, was admitted to hospital with a three-day history of worsening shortness of breath, productive cough with yellow sputum, and pain in her lower back and both lower limbs. She denied any gastrointestinal symptoms.

In terms of her sickle cell anemia, the patient reported two previous sickle cell crises. The first in Grenada, in 2002, when she was plasma exchanged and the second, five months previously in Barbados, when she was transfused one unit of blood. She also had a history of pigmented gallstones. She was taking regular folic acid. Her vaccinations were not up-to-date and no prophylactic penicillin was being taken.

On examination, the patient was dehydrated, febrile at 38.5°C, tachycardic and desaturating on room air. She was clinically jaundiced. A systolic flow murmur was noted on examination of her cardiovascular system. On auscultation of her chest, there was reduced air entry and crepitations at the right base. Her abdominal examination revealed a mildly tender, enlarged liver. Her lower back and both thighs were tender on palpation.

Laboratory results showed a hemoglobin of 6.7 g/dL (her baseline hemoglobin was 7 g/dL); mean cell volume 87.8 fl; white cell count 18.7 × 10^9^/L; neutrophils 13.6 × 10^9^/L; platelets 81.0 × 10^9^/L; C-reactive protein > 250 mg/L; creatinine kinase 10 U/L. Chest radiography showed a globular heart, moderate bilateral effusions, sickle cell bony changes but no focal consolidation.

Our patient's chest crisis was managed with analgesia, hydration, and continuous positive airway pressure (CPAP). Intravenous amoxicillin (500 mg every eight hours) and clarithromycin (500 mg every 12 hours) were prescribed to cover a possible chest infection. Despite this, her clinical condition continued to deteriorate and her hemoglobin dropped to 5.4 g/dL. It was then decided to perform an exchange transfusion.

On the second hospital day, *Salmonella agbeni *was grown from blood cultures and intravenous ceftriaxone (2 g every 24 hours) commenced. The patient continued to have persistent temperature spikes of > 38.5°C despite a two-week course of ceftriaxone. For enhanced intra-cellular penetration, ceftriaxone was then changed to oral ciprofloxacin (500 mg every 12 hours), to which the organism was also sensitive. Repeat blood cultures and stool cultures to assess carrier status were negative.

**Figure 1:**
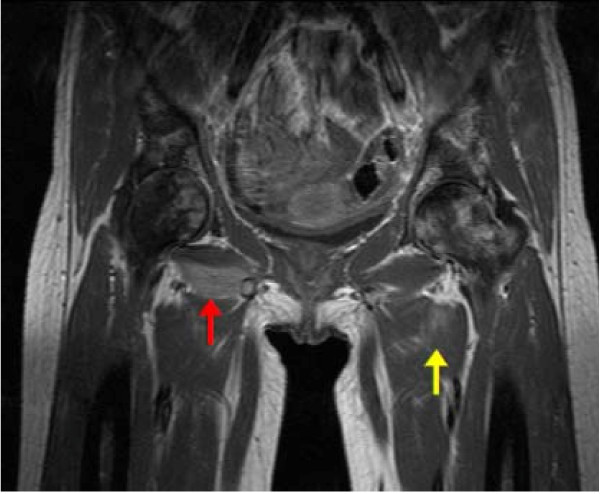
**Coronal T1-weighted MRI of the pelvis and hips - initial imaging**. Left obturator externus and adductor brevis muscles (yellow arrow), and the right obturator externus, iliacus and iliopsoas muscles (red arrow).

After seven days of ciprofloxacin our patient developed limited hip extension on the right with persistently raised inflammatory markers: C-reactive protein 237 mg/L; white cell count 16.2 × 10^9^/L; neutrophils 12.3 × 10^9^/L. In order to ascertain a focus of infection, plain radiography and a bone scan of her hips and pelvis were performed. The former demonstrated chronic avascular necrosis and the latter increased vascularity around the right hip. It was suggested this may represent infection and a magnetic resonance imaging (MRI) scan of the hips and pelvis was performed. This revealed the diagnosis of pyomyositis, with particular involvement of the left obturator externus and adductor brevis muscles (yellow arrow), and the right obturator externus, iliacus and iliopsoas muscles (red arrow) (Figure 1).

**Figure 2:**
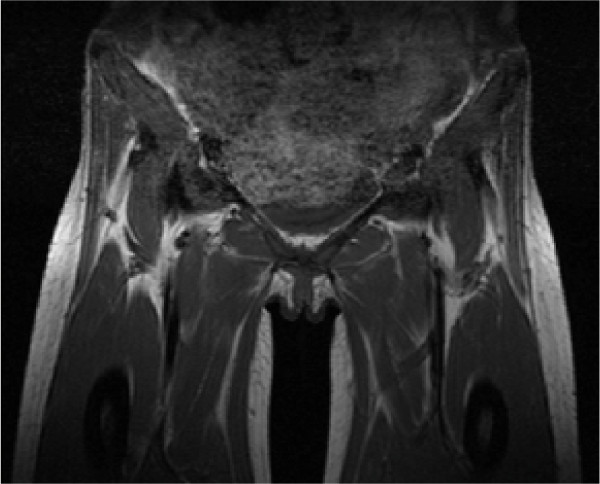
**Coronal T1-weighted MRI of the pelvis and hips - repeat imaging**.

Both orthopedic and radiology teams were consulted and it was decided there was no collection that could be drained either surgically or radiologically. As the source of infection was most likely to be gastrointestinal, a combination of intravenous ceftriaxone (2 g every 24 hours) and metronidazole (400 mg every eight hours) was prescribed to cover the possibility of a polymicrobial infection. Our patient was examined after a total of six weeks of intravenous antibiotic therapy and was found to have no residual functional limitations, her C-reactive protein had normalized and resolution was noted on repeat MRI (Figure 2).

## Discussion

Pyomyositis is classically an infection of the tropics and primarily affects healthy children and young adults [4]. In temperate countries most patients are immunocompromised or have other serious underlying conditions [2,4]. Our patient was originally from the tropics and was immunosuppressed with sickle cell disease.

The primary diagnosis for her leg and hip pain was ongoing bony crisis in sickle cell anemia, despite the blood culture growing *S. agbeni *at the start of admission, and she was treated accordingly. This approach, however, caused delay in diagnosing and subsequently treating pyomyositis. A high index of suspicion is needed to make the diagnosis of pyomyositis because clinical signs are not always specific and muscle pain and fever are both commonplace in sickle cell crises.

The tools for diagnosing pyomyositis include radiological investigation and microbiological cultures. MRI not only confirms the diagnosis, but also delineates the extent of infection and establishes any other entities such as abscess formation or osteomyelitis [5].

Pyomyositis is a result of hematogenous spread and bacteremia has been reported in up to 31% cases [6]. *S. agbeni *grown in blood cultures gave us the likely causative organism in this case. Salmonella is not a common pathogen of muscle infections but should be considered in patients with underlying disease. There is a significant mortality rate associated with Salmonella muscle infections compared to the most common cause of pyomyositis, *Staphylococcus aureus *[3].

Treatment comprises a combination of antibiotics and surgical or percutaneous drainage as appropriate. There are no set guidelines for the duration of antimicrobial treatment and this should be tailored to clinical, laboratory and radiological improvement. The evidence shows that three to four weeks of parenteral therapy is usually sufficient, however in patients with more complicated infection a longer course may be necessary [2,6].

## Conclusions

We have reported the case of a 44-year-old Afro-Caribbean woman with homozygous sickle disease who developed Salmonella pyomyositis of her hip and pelvic muscles. Patients with sickle cell anemia have increased susceptibility to infection and pyomyositis should be considered in sickle cell patients who present with persistent fever, localized muscle pain and muscle tenderness. The availability of MRI can facilitate early diagnosis and prevent complications. Treatment includes appropriate antibiotics and drainage if indicated.

## Abbreviations

CPAP: continuous positive airway pressure; HbSS: homozygous sickle cell disease; MRI: magnetic resonance imaging.

## Consent

Written informed consent was obtained from the patient for publication of this case report and any accompanying images. A copy of the written consent is available for review by the Editor-in-Chief of this journal.

## Competing interests

The authors declare that they have no competing interests.

## Authors' contributions

VKW, MEL and JAM contributed to the conception and design of the manuscript. VKW was involved in the literature research and review, as well as the manuscript preparation and manuscript review. MEL helped draft part of the manuscript and contributed to the review of the manuscript. TDT reviewed the medical imaging and helped with the manuscript review. JAM supervised and contributed to the manuscript review. All the authors have reviewed and approved the final manuscript.
